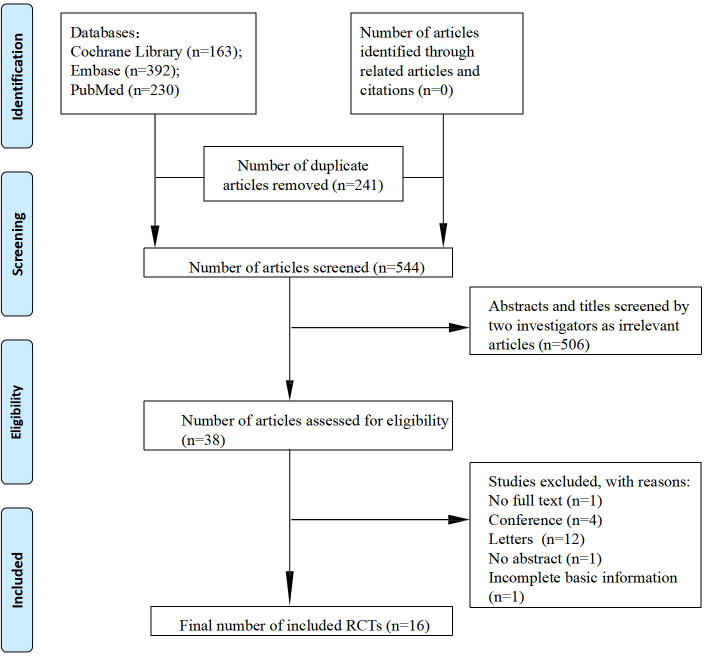# Efficacy and safety of Janus kinase inhibitors in the treatment of psoriasis and psoriatic arthritis: An analysis of evidence from 2014 to 2022

**DOI:** 10.1016/j.heliyon.2025.e42084

**Published:** 2025-01-28

**Authors:** Jiao Wang, Tian Ma, Xiaoying Sun, Liu Liu, Seokgyeong Hong, Huijung Cha, Miao Zhang, Jiale Chen, Naixuan Lin, Bin Li, Xin Li

**Affiliations:** a Department of Dermatology, Yueyang Hospital of Integrated Traditional Chinese and Western Medicine, Shanghai University of Traditional Chinese Medicine, Shanghai, 200437, China; b Institute of Dermatology, Shanghai Academy of Traditional Chinese Medicine, Shanghai, 201203, China; c Department of Dermatology, Shanghai Skin Disease Hospital, Shanghai, 200443, China

**Keywords:** Janus kinase (JAK) inhibitors, Psoriasis (PSO), Psoriatic arthritis (PsA), Randomized controlled study, Meta-analysis

## Abstract

**Background:**

The efficacy and safety of Janus kinase (JAK) inhibitors as novel therapeutic agents for psoriasis (PSO) and psoriatic arthritis (PsA) have not yet been systematically evaluated.

**Methods:**

Randomized controlled studies (RCTs) assessing JAK inhibitors to treat PSO or PsA published before September 1, 2022, were searched in the PubMed, Embase, and Cochrane Library databases. The Psoriasis Area and Severity Index (PASI) 75 and American College of Rheumatology (ACR) 50 were established as primary outcome indicators of PSO and PsA, respectively. Adverse events (AEs) were classified according to eight systems of the human body.

**Results:**

We included 16 relevant RCTs involving 4,936 patients with psoriasis, with interventions performed using different doses of JAK inhibitors. Except in one study, all patients received a placebo or vehicle control. JAK inhibitors were administered topically in three studies and orally administered in the remaining studies. Meta-analysis revealed that oral administration of JAK inhibitors significantly improved PASI 75 in patients with PSO (risk ratio [RR] 3.29; 95 % confidence interval [CI] [2.37, 4.57]), and topical application was not effective (RR 1.45, 95 % CI [0.97, 2.15]). JAK1 and JAK3 inhibitors were more effective than JAK1/JAK 2 inhibitors for PASI 75. JAK1 inhibitors elicited high response rates in improving ACR 50 in PsA (RR 2.95, 95 % CI [1.63, 5.35]) and achieved significant results at week 16 of treatment (RR 2.95, 95 % CI [1.63, 5.35]). Oral JAK inhibitor-induced AEs were mainly related to the circulatory system (RR 3.09, 95 % CI [1.42, 6.75]).

**Conclusions:**

Oral JAK inhibitors improved clinical symptoms in patients with PSO. Tofacitinib, a JAK1 and JAK3 inhibitor, has a high response rate. Filgotinib, a JAK1 inhibitor, markedly enhanced the response rate and improved ACR scores in patients with PsA, and the best effect was achieved with 16-week oral administration.

## 1 Introduction

Psoriasis (PSO) is a chronic immune-mediated inflammatory skin disorder typically characterized by keratinocyte hyperproliferation. Approximately 2–3% of the global population suffers from this condition, which has been associated with genetics, infections, the environment, and metabolic conditions [1]. The comorbidity of PSO with cardiovascular diseases, lipid metabolism, and chronic obstructive pulmonary diseases is well established and can markedly affect the health and quality of life of patients [2]. Individuals with PSO reportedly attempt suicide 32 % more frequently than those without PSO [3]. Currently, there is no known cure for this disease owing to its complex nature. PSO treatment options, such as cyclosporine, methotrexate, and phototherapy, are only suitable for mild-to-moderate cases. In recent years, the use of biological agents, such as anti-interleukin (IL)-17, anti-IL-23, and anti-tumor necrosis factor (TNF)-α, has demonstrated considerable success in treating patients with severe PSO [4]; however, these agents can induce adverse events (AEs), such as infection. Accordingly, given the possibility of adverse reactions, such as infection, and the potential for relapse following treatment discontinuation, biological agents are not well accepted [5]. Therefore, there is an urgent need to develop effective, safe, and convenient therapeutic strategies.

Janus kinase (JAK) inhibitors have been approved as small-molecule targeted therapies for various dermatological conditions, including alopecia areata, vitiligo, and atopic dermatitis (AD). The JAK family comprises four isoforms: JAK1, JAK2, JAK3, and tyrosine kinase 2 (TYK2). JAK activation generates tyrosine phosphorylation sites for docking signal transducer and activator of transcription (STAT) with bound receptors [6]. JAK/STAT is an inflammatory signaling pathway involved in the transduction of various cytokine signals and is closely associated with several important biological functions, such as cell growth, differentiation, apoptosis, and immune regulation. Tofacitinib, ruxolitinib, baricitinib, and filgotinib are the most commonly used JAK inhibitors [7]. Tofacitinib is a specific JAK1 and JAK3 inhibitor. Tofacitinib was initially approved by the U.S. Food and Drug Administration (FDA) to treat moderately to severely active rheumatoid arthritis [8]. Ruxolitinib targets JAK1 and JAK2 and has been approved to treat intermediate-to high-risk myelofibrosis. Furthermore, ruxolitinib has been found to reduce dendritic cell (DC) activation and migration, inhibit inflammation, and modulate immune response [9]. Baricitinib, a JAK1 and JAK2 inhibitor, was the first JAK inhibitor licensed for use in patients with moderate-to-severe AD in the European Union. Baricitinib can substantially inhibit helper T 1 (Th1) and Th17-related cytokines [10]. Filgotinib, an oral drug targeting the JAK1-mediated signaling pathway, is primarily used to treat inflammatory arthritis and ulcerative colitis. Filgotinib was shown to dose-dependently reduce JAK1-associated IL-6-induced STAT1 phosphorylation in human whole blood. Furthermore, it primarily suppressed JAK1 activity *in vitro*. The most common filgotinib-induced adverse reactions include dizziness, gastrointestinal reactions, and minor infections (including upper respiratory and urinary tract) [11]. Other JAK1 inhibitors included abrocitinib, itacitinib, and solcitinib. Abrocitinib, which targets adenosine triphosphate binding sites, was approved by the U.S. FDA in 2022 to treat refractory moderate-to-severe AD and is the first JAK1 inhibitor for AD worldwide. In a pharmacokinetic study undertaken in healthy volunteers, abrocitinib had a faster absorption time at low doses than at high doses, and each metabolite exhibited a greater renal clearance [12,13]. Itacitinib reportedly signals inflammatory factors such as interferon (IFN)-γ and IL-6 in cytokine release syndrome and can prevent the induction of lymphocytopenia, a side effect associated with JAK1/JAK2 inhibitors [14]. Solcitinib is a highly selective and irreversible JAK1 inhibitor that has been used to treat plaque psoriasis [15].

In recent clinical research, small-molecule drugs have elicited unexpected therapeutic benefits in the treatment of moderate-to-severe PSO; however, these agents can induce AEs, including infection, heart failure, hyperlipidemia, and other side effects [7]. In this systematic review, we identified and included high-quality randomized controlled trials (RCTs) to assess the efficacy of JAK inhibitors in treating psoriasis and AEs that merit clinical attention.

## 2 Methods

### 2.1 Literature search strategy

To assess the efficacy and safety of JAK inhibitors in PSO, we followed the Preferred Reporting Items for Systematic Reviews and Meta-Analyses guidelines to search PubMed, Embase, and the Cochrane Library and identified randomized controlled trials (RCTs) assessing JAK inhibitors for treating PSO**.** The search strategy was as follows: we used a combination of subject headings and free words (psoriasis [MeSH]) and (JAK inhibitors [MeSH]). The search period extended from database initiation to September 1, 2022, with no language restrictions. The retrieval strategy is illustrated in Fig. S1. The process of searching and screening literature was carried out independently by two authors (S.H. and H.C.). In case of disagreements, a third author (J.W.) intervened to reach a consensus. Studies eligible for this systematic review were required to meet the following criteria: (a) an RCT assessing JAK inhibitors for the treatment of psoriasis, (b) PSO or psoriatic arthritis (PsA), and (c) at least one measure of efficacy or safety.

### 2.2 Data extraction

Two authors (J.W. and T.M.) independently extracted the basic characteristics of the included studies, such as the study drug, target, sample size, intervention methods, treatment time, and outcomes. Different values were used for the meta-analysis of all continuous variables to obtain more reliable conclusions. If a study examined multiple cohort doses of JAK inhibitors, it was classified as multiple studies.

### 2.3 Outcomes

#### 2.3.1 Efficacy

The Psoriasis Area and Severity Index (PASI) 75 was used as the primary outcome measure in patients with PSO. Secondary outcomes included PASI 90, PSAI 50, Dermatology Life Quality Index (DLQI), body surface area (BSA), physician's global assessment (PGA-C), itch severity item (ISI) score, and patient global assessment (PtGA). The ACR 50 response was used as the primary outcome for PsA; ACR 20, ACR 70, Short Form-36 questionnaire, version 2 (SF-36), and disease activity in psoriatic arthritis (DAPSA) were used as secondary outcomes.

#### 2.3.2 Safety

All AEs were classified according to the eight major systems of the human body: exercise, nervous, endocrine, circulatory, respiratory, digestive, and urinary. No reproduction-related adverse reactions were observed. Skin-related and other AEs were also included. The AE data for each study are presented in Table S1.

### 2.4 Risk of bias assessment

The Cochrane Collaboration tool is well-recognized as the best tool for assessing the risk of bias in RCTs. Two reviewers (X.Y.S. and L.L.) independently assessed the risk of bias in the included studies, and when the results were inconsistent, both parties negotiated and reached a consensus.

### 2.5 Statistical analysis

Data analyses were performed using RevMan software (version 5.3). Continuous variables are presented as mean differences (MD) and 95 % confidence intervals (CI), while dichotomous variables are presented as risk ratios (RRs) and 95 % CIs. I^2^ was used to assess the heterogeneity of analyzed results, and a random-effects model was applied if the heterogeneity was greater than 50 %.

## 3 Results

### 3.1 Search results and trial characteristics

We retrieved 785 relevant articles from PubMed, Embase, and Cochrane Library databases and subsequently deleted 241 duplicate articles. After reading the titles and abstracts, 506 non-relevant articles were excluded from the analysis. The remaining 38 articles comprised 4 conferences, 12 letters, 1 no-abstract, 1 incomplete basic information and 1 unavailable full-text article. Finally, 16 RCTs involving 4,936 patients were included in the present study. Thirteen articles examined JAK inhibitors for PSO [[16], [17], [18], [19], [20], [21], [22], [23], [24], [25], [26], [27], [28]], and three examined their application in PsA [[29], [30], [31]]. Seven JAK inhibitors were evaluated: tofacitinib, peficitinib, baricitinib, ruxolitinib, solcitinib, abrocitinib, and filgotinib. JAK inhibitors were topically administered in three studies [16,18,22] and were administered orally in the remaining reports. All studies were placebo-controlled, except for one [31], in which the control group was a positive control. AEs were not mentioned in six study outcome indicators [[18], [19], [20],^22^,^29^,^30^]. Except for one study [31], all other studies provided registration numbers. Detailed characteristics of the included studies are presented in Table S2.

### 3.2 Study quality

The results of the bias analysis are listed in Table S3. Most studies were low risk, but individual studies showed a high risk. Ports et al. [18] used ISI and patient satisfaction with study medication as outcome indicators, although no relevant data were identified in the article; therefore, we classified item-selective outcome reporting as high-risk. Sharma et al. [31] used open labels; hence, item allocation concealment, blinding of participants and personnel, and blinding of outcome assessment were judged as high-risk, along with blinding of participants and personnel and blinding of outcome assessment. Risk indicators not described in this study were defined as uncertain risks.

### 3.3 Efficacy outcomes

#### 3.3.1 Primary efficacy outcome measure for PSO

##### 3.3.1.1 PASI 75

Based on the meta-analysis results, oral administration of JAK inhibitors improved PASI 75 when compared with the placebo group (RR 3.29, 95 % CI [2.37, 4.57], I^2^ = 61 %), whereas topical application exerted no therapeutic effect (RR 1.45, 95 % CI [0.97, 2.15], I^2^ = 0 %). JAK inhibitors had a rapid onset of action and could improve PASI 75 response rates in approximately four weeks (RR 3.76, 95 % CI [2.38, 5.95], I^2^ = 11 %) (Table 1).

**Table 1:**
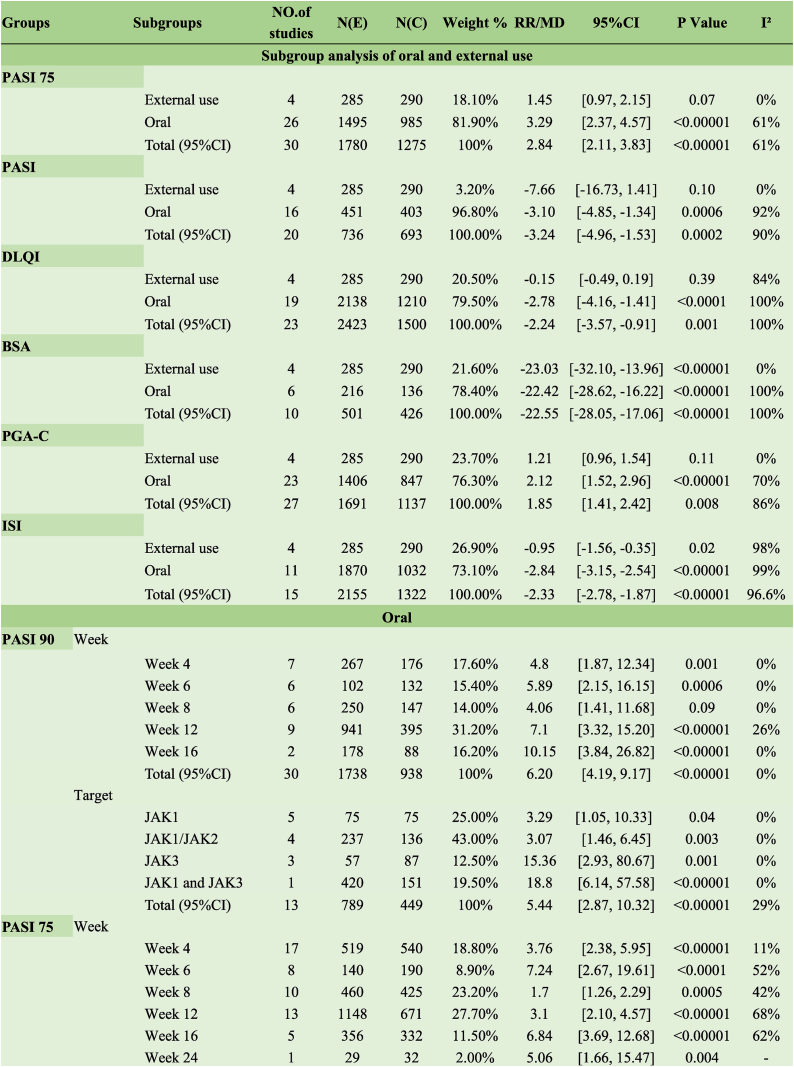

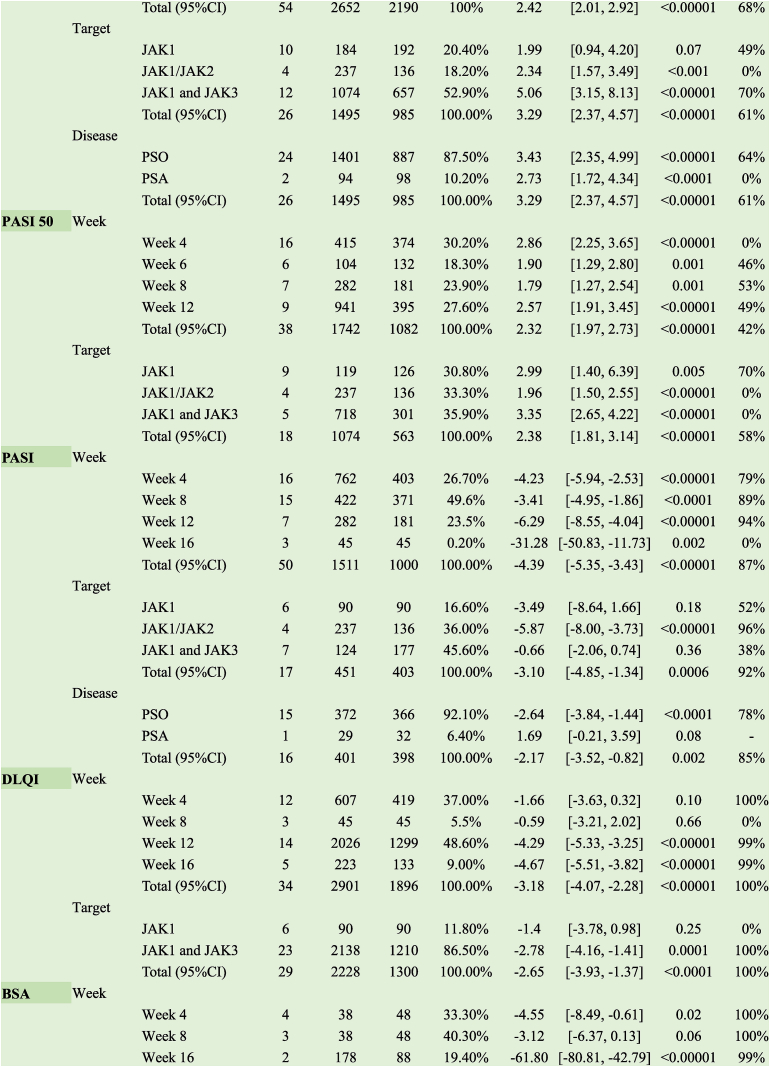

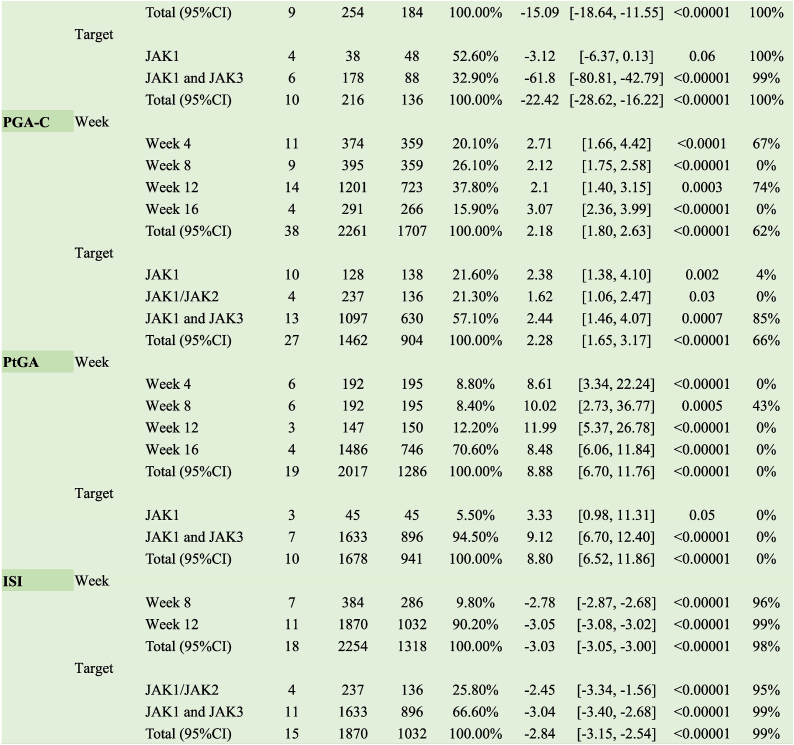
Clinical effects of JAK inhibitors of psoriasis.

#### 3.3.2 Secondary efficacy outcome measures for PSO

##### 3.3.2.1 PASI 90

According to the subgroup analysis, oral JAK inhibitors with different targets increased PASI 90 response rates after 4, 6, 12, and 16 weeks of treatment when compared with the placebo (RR 6.20, 95 % CI [4.19, 9.17], I^2^ = 0 %) (Table 1).

##### 3.3.2.2 PASI 50

Subgroup analysis revealed that oral JAK inhibitors with different targets increased PASI 50 response rates after 4, 6, 8, and 12 weeks of treatment when compared with the placebo (RR 2.32, 95 % CI [1.97, 2.73]; I^2^=42 %). JAK inhibitors had a rapid onset of action, promptly increasing PASI 50 response rates by week 4 of treatment (RR 2.86, 95 % CI [2.25, 3.65], I^2^ = 0 %) (Table 1).

##### 3.3.2.3 PASI

Likewise, a meta-analysis of continuous variables for PASI scores showed that oral JAK inhibitors significantly improved PASI scores when compared with the placebo (MD −3.10, 95 % CI [−4.85, −1.34], I^2^ = 92 %); however, this effect was not observed upon topical application (MD −7.66, 95 % CI [−16.73, 1.41], I^2^ = 0 %). Oral inhibitors targeting JAK1/JAK2 improved PASI scores (MD −5.87, 95 % CI [−8.00, −3.73], I^2^ = 96 %), whereas inhibitors targeting JAK1 (MD −3.49, 95 % CI [−8.64, 1.66], I^2^ = 52 %) or JAK1 and JAK3 (MD −0.66, 95 % CI [−2.06, 0.74], I^2^ = 38 %) failed to improve PASI scores (Table 1).

##### 3.3.2.4 DLQI

Compared with the placebo, oral JAK1 and JAK3 inhibitors significantly reduced DLQI scores in patients (MD −2.78, 95 % CI [−4.16, −1.41], I^2^ = 100 %); however, this effect was not observed following topical application (MD −0.15, 95 % CI [−0.49, 0.19], I^2^ = 84 %). Subgroup analysis revealed the absence of any effect on DLQI when the treatment duration was 4 (MD −1.66, 95 % CI [−3.63, 0.32]; I^2^ = 100 %) and 8 weeks (MD, −0.59 95 % CI [−3.21, 2.02], I^2^ = 0 %), and patients' DLQI scores were significantly reduced when the dosing duration reached 12 weeks (MD −4.29, 95 % CI [−5.33, −3.25]; I^2^ = 99 %) and 16 weeks (MD−4.67, 95 % CI[−5.51,−3.82], I^2^ = 99 %) (Table 1).

##### 3.3.2.5 BSA

Subgroup analysis showed that oral JAK inhibitors significantly improved BSA. JAK1 and JAK3 inhibitors provided superior BSA improvement after 16 weeks of treatment (MD −61.80, 95 % CI [−80.81, −42.79]; I^2^ = 99 %) (Table 1).

##### 3.3.2.6 PGA-C

According to the subgroup analysis, oral administration of JAK inhibitors with distinct targets improved PGA-C at treatment greater than 4 weeks, while topically administered JAK inhibitors did not impact PGA-C (RR 1.21, 95 % CI [0.96, 1.54] ) (Table 1).

##### 3.3.2.7 ISI score

Based on the subgroup analysis, oral or topical JAK inhibitors with different targets significantly improved patients' ISI scores (MD −2.33, 95 % CI [−2.78, −1.87], I^2^ = 96.6 %). The best results achieved 8 or 12 weeks post-treatment when taken orally (MD −3.03, 95 % CI [−3.05, −3.00], I^2^ = 98 %) (Table 1).

##### 3.3.2.8 PtGA

Subgroup analysis showed that oral JAK1 and JAK3 inhibitors significantly improved PtGA (RR 9.12, 95 % CI [6.70, 12.40], I^2^ = 0 %), with optimal efficacy achieved after 4 weeks of treatment (RR 8.61, 95 % CI [3.34, 22.24], I^2^ = 0 %) (Table 1).

#### 3.3.3 Primary efficacy outcome measure for PsA

##### 3.3.3.1 ACR 50

JAK1 inhibitors increased the response rate of ACR 50 in patients with PsA (RR 2.95, 95 % CI [1.63, 5.35]); JAK1 and JAK3 inhibitors did not exhibit the same effect (RR 1.24, 95 % CI [0.55, 2.79]). The duration of treatment at 4 (RR 3.31, 95 % CI [0.36, 30.08]) and 12 (RR 1.24, 95 % CI [0.55, 2.79]) weeks did not affect the response rate to ACR 50, and effects were observed when dosing reached 16 weeks (RR 2.95, 95 % CI [1.63, 5.35]) (Table 2).

**Table 2:**
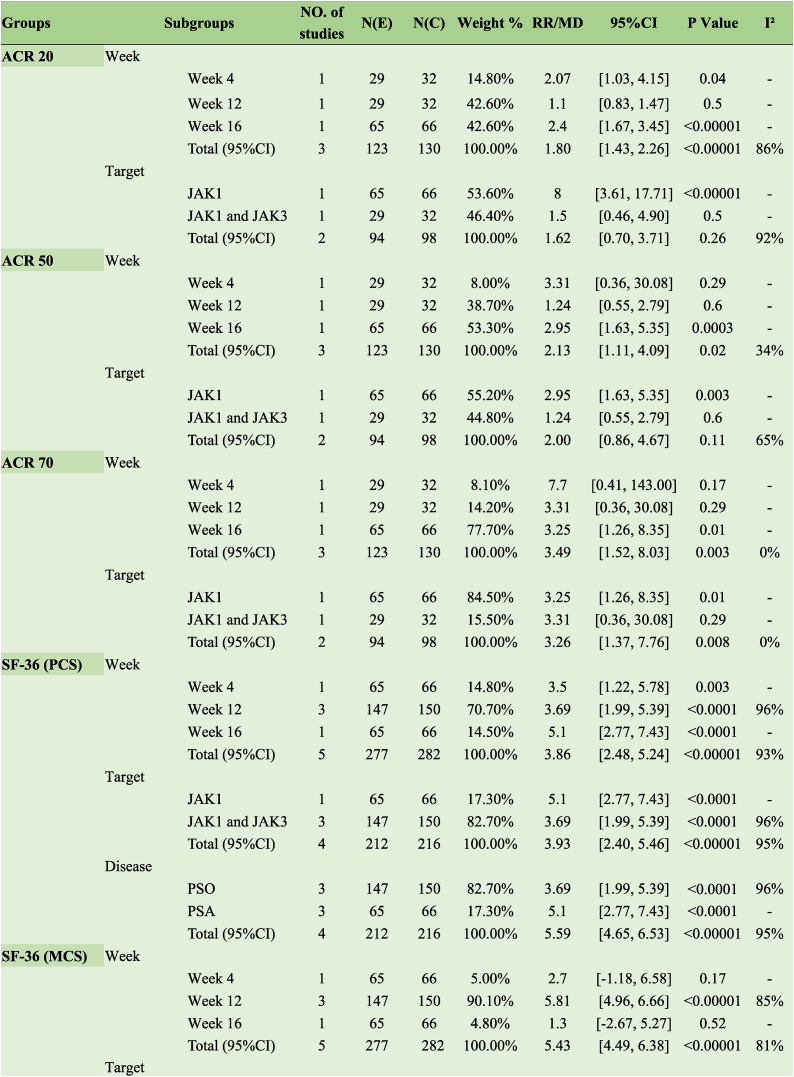

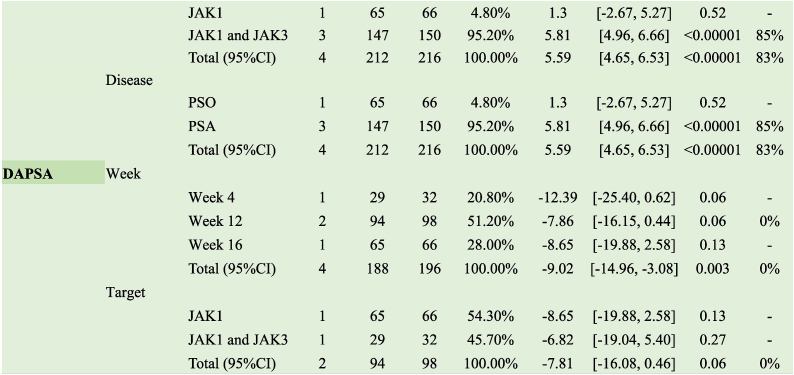
Clinical effects of JAK inhibitors of psoriatic arthritis.

#### 3.3.4 Secondary efficacy outcome measures for PsA

##### 3.3.4.1 ACR 20

Inhibitors targeting JAK1 increased the ACR 20 response rate in patients with PsA (RR 8.00, 95 % CI [3.61, 17.71]), whereas those targeting JAK1 and JAK3 did not elicit the same effect (RR 1.50, 95 % CI [0.46, 4.90]). A 12-week treatment duration (RR 1.10, 95 % CI [0.83, 1.47]) did not affect the ACR 50 response rate, with effects only observed when dosing reached 16 weeks (RR 2.40, 95 % CI [1.67, 3.45]) (Table 2).

##### 3.3.4.2 ACR 70

JAK1 inhibitors increased the ACR 50 response rate in patients with PsA (RR 3.25, 95 % CI [1.26, 8.35]); however, JAK1 and JAK3 inhibitors did not exert the same effect (RR 3.31, 95 % CI [0.36, 30.08]). Treatment durations of 4 (RR 7.7, 95 % CI [0.41, 143.00]) and 12 weeks (RR 3.31, 95 % CI [0.36, 30.08]) did not affect the ACR 70 response rate, with effects observed only when dosing reached 16 weeks (RR 3.25, 95 % CI [1.26, 8.35]) (Table 2).

##### 3.3.4.3 SF-36

JAK inhibitors with different targets significantly improved SF-36 (PCS [the Physical Component Summary]) (MD 3.93, 95 % CI [2.40, 5.46], I^2^ = 95 %) and exerted therapeutic effects after 4, 12, and 16 weeks of treatment (MD 3.86, 95 % CI [2.48, 5.24]; I^2^ = 93 %). However, only inhibitors targeting JAK1 and JAK3 improved SF-36 (MCS [the Mental Component Summary]), with the best results achieved at 12 weeks of treatment (MD 5.81, 95 % CI [4.96, 6.66], I^2^ = 85 %) (Table 2).

##### 3.3.4.4 DAPSA

Subgroup analysis revealed no improvement in DAPSA scores following treatment with either oral or topical JAK inhibitors (Table 2).

### 3.4 Safety outcomes

**Fig. 1:**
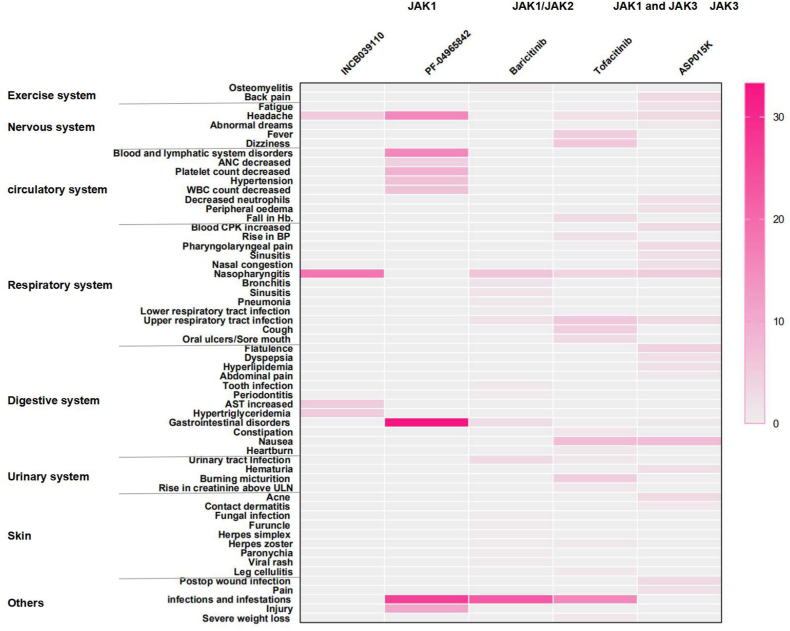
Heat map of different types of AEs. AEs, adverse events. Red areas indicate a greater relative probability of occurrence, and lighter-colored areas indicate a slight or null relative probability of occurrence.

Based on the findings of the meta-analysis, oral JAK inhibitors could induce circulatory AEs (RR 3.09, 95 % CI [1.42, 6.75], I^2^ = 0 %), including neutropenia, decreased hemoglobin, and increased blood pressure. Oral JAK inhibitors can cause adverse reactions in other systems, such as the respiratory and exercise systems, with no statistically significant differences. Topical JAK inhibitors did not induce any significant AEs (Table 3). Fig. 1 presents a heatmap to visualize the rates of AEs caused by JAK inhibitors with different targets.

**Table 3:**
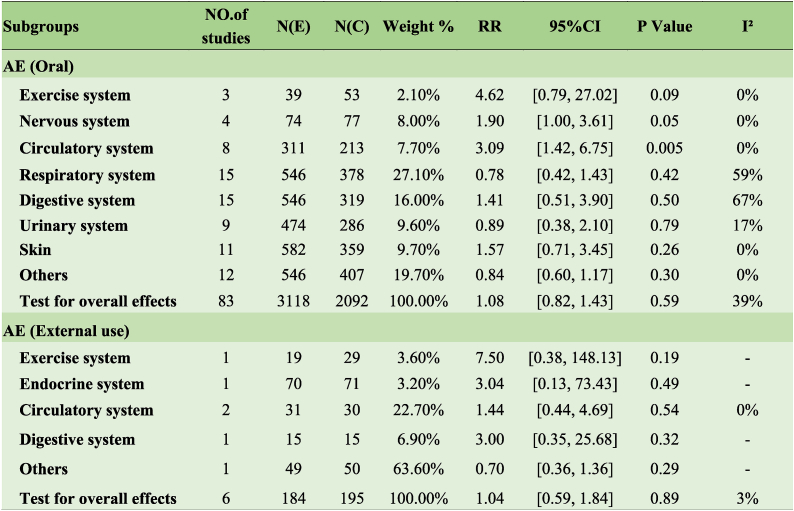
Adverse effects of Clinical effects of JAK inhibitors of psoriasis and psoriatic arthritis.

## 4 Discussion

Our systematic review included 16 high-quality RCTs that evaluated the efficacy and safety of JAK inhibitors with different targets, including tofacitinib, baricitinib, itacitinib, peficitinib, ruxolitinib, abrocitinib, and filgotinib, in PSO and PsA.

In the present study, our findings revealed that JAK inhibitors exhibited higher efficacy when administered orally than topically. Compared with the placebo, almost all orally administered JAK inhibitors showed improved efficacy in treating PSO, as determined by superior scores for PASI, DLQI, BSA, PGA-C, PtGA, and ISI, along with efficacy indicators of PsA, such as ACR, SF-36 (PCS), SF-36 (MCS), and DAPSA. Additionally, JAK inhibitors can rapidly initiate therapeutic effects against PSO, increasing the response rates of PASI, PGA-C, PtGA, and other indicators within 4 weeks of administration, accompanied by improvements in the patient's quality of life, lesion area, and itching. Treatment with JAK inhibitors elicited superior outcomes when administered for at least 16 weeks. Inhibitors targeting JAK1 and JAK3 were the most effective in treating PSO, substantially improving all efficacy indicators, followed by JAK1/JAK2 inhibitors. JAK1 inhibitors did not affect PASI 75 response rates in patients, while DLQI, BSA, and PtGA had a marginal effect on PASI 90 and PASI 50 response rates. Subgroup analysis showed that topical JAK inhibitors slightly improved the DLQI and ISI, with no statistical differences in other indicators. According to the results of our meta-analysis, JAK1-targeted inhibitors markedly improve the ACR and DAPSA in PsA, whereas JAK1 and JAK3 inhibitors exerted the opposite effect and delayed therapeutic effects (16 weeks). Accordingly, we recommend oral JAK1 and JAK3 inhibitors to treat PSO in clinical practice and oral JAK1 inhibitors to treat PsA, preferably for 16 weeks. Our study found that the negative effects of topical JAK inhibitors were statistically insignificant, despite the findings of clinical trials on JAK inhibitors to treat PSO, suggesting that both oral and topical administration can result in skin conditions such as infections, gastrointestinal disorders, and herpes zoster. Regarding the adverse effects of oral JAK inhibitors, substantial AEs were only observed in the circulatory system, including elevated blood creatine phosphokinase, reduced neutrophil counts, and peripheral edema. Adverse responses in the other systems, including the skin, were statistically insignificant.

**Fig. 2:**
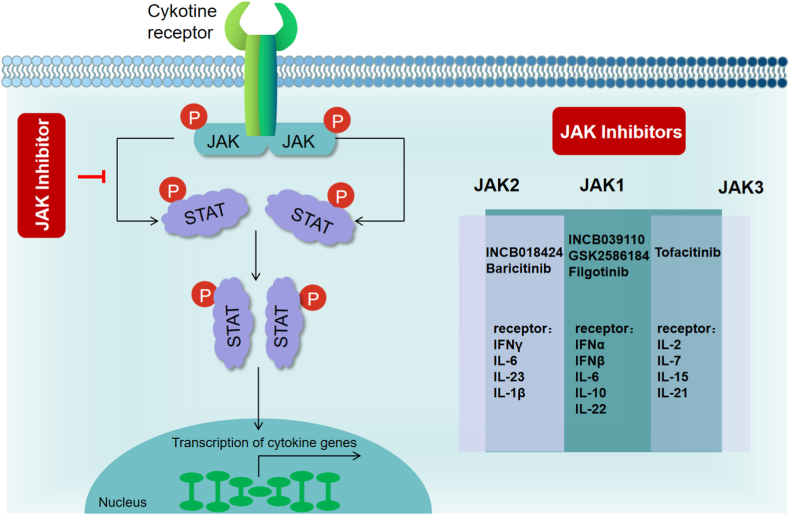
The mechanism of action of JAK inhibitors in the treatment of PSO and PsA JAK, Janus kinase; PSO, psoriasis; PsA, psoriatic arthritis, STAT, signal transducer and activator of transcription. Different cytokines bind to their receptors and phosphorylate JAK and STAT, which are transcribed into inflammatory factors after entering the nucleus to initiate an inflammatory cycle. JAK inhibitors block the phosphorylation of JAK and STAT, thereby inhibiting inflammatory cytokine production.

JAK inhibitors, a new class of targeted synthetic disease-modifying anti-rheumatic drugs, have been approved to treat numerous immune-mediated inflammatory disorders, including PSO. JAK inhibitors reduce inflammation by transducing signals from various cytokine receptors, such as type I (IFN-α/β) and type II (IFN-γ). Expression levels of STAT1 and STAT3 were found to be considerably elevated in psoriatic lesions when compared with those in normal skin [32]. STAT1 predominantly utilizes the JAK1/JAK2-dependent pathway to transduce type I and II IFN signals. STAT1 activation induces the production of inflammatory mediators and stimulates Th17 cells proximally [33]. JAK1, JAK2, and TYK2 are primary activators of STAT3. Through IL-6-induced JAK1/JAK2 or JAK1/TYK2 signaling, STAT3 increases keratinocyte proliferation and development in Th17 cells [34]. During PSO, when JAK1 and JAK2 copolymerize, the inhibitor mainly acts on the receptors of IFN-γ, IL-6, IL-23, and IL-1β, while the JAK1 inhibitor mainly acts on the receptors of type I IFN, IL-6, IL-10, and IL-22. In PsA, when JAK1 copolymerizes with JAK3, the inhibitor predominantly acts on IL-2, IL-7, IL-15, and IL-21 receptors [35,36]. Therefore, JAK inhibitors may be viable treatment options for PSO and PsA. Fig. 2 presents a diagram illustrating the underlying mechanism of action of JAK inhibitors in the treatment of PSO and PsA.

As the highest level of evidence, the present meta-analysis confirmed the safety and efficacy of JAK inhibitors for treating immune-mediated diseases. JAK inhibitors have been investigated to treat alopecia areata, and oral JAK inhibitors can substantially maintain hair regrowth [37]. A previous meta-analysis has confirmed that JAK inhibitors can effectively and safely treat AD [38]. Furthermore, a recent meta-analysis assessing the safety of JAK inhibitors in immune-mediated diseases revealed that JAK inhibitors may increase the risk of herpes zoster, although the incidence of other AEs did not increase [7]. This finding differed from that of the AE analysis. This discrepancy could be attributed to the categorization of AEs based on the different systems of the human body in the present study. Our study revealed statistically significant AEs in the circulatory system, suggesting that monitoring of circulatory system-related indicators should be expanded during clinical drug administration.

The present meta-analysis evaluated 15 indicators, which facilitated a comprehensive analysis of the efficacy of JAK inhibitors. Additionally, we included high-quality RCTs and employed differences in continuous variables to provide more credible and objective results. Nonetheless, this study has certain limitations, as evidenced by the following factors. First, only one study provided data on the ACR for PsA, thereby reducing the credibility of the obtained results. Second, the articles on TYK2 were letters; therefore, they were not included in our analysis, which may have resulted in incomplete results. Third, only one study investigated itacitinib, peficitinib, ruxolitinib, and abrocitinib; therefore, the conclusions may have been highly biased. Finally, not all of the included studies evaluated AEs, and the included patients were not excluded from the underlying disease. Future studies with larger sample sizes and higher quality are required to identify optimal JAK inhibitors to treat PSO and PsA.

## 5 Conclusions

In conclusion, the results of our meta-analysis suggest that oral JAK inhibitors are more effective than topical JAK inhibitors. In patients with PSO, JAK1 and JAK3 inhibitors were superior to JAK1/JAK2 inhibitors, with the least effectiveness demonstrated by JAK1 inhibitors. Notably, a pronounced efficacy was observed after 16 weeks of treatment. JAK1 inhibitors were markedly effective against PsA, demonstrating initial efficacy after 16 weeks of treatment. JAK inhibitors can also cause adverse circulatory events. Additional high-quality multicenter prospective studies with larger sample sizes are needed to clarify the safety of JAK inhibitors.

## CRediT authorship contribution statement

**Jiao Wang:** Writing – original draft, Visualization, Validation, Supervision. **Tian Ma:** Project administration, Methodology. **Xiaoying Sun:** Data curation. **Liu Liu:** Investigation. **Seokgyeong Hong:** Software. **Huijung Cha:** Methodology. **Miao Zhang:** Formal analysis. **Jiale Chen:** Project administration. **Naixuan Lin:** Visualization, Validation, Conceptualization. **Bin Li:** Supervision. **Xin Li:** Writing – review & editing, Supervision, Conceptualization.

## Ethics approval and consent to participate

Not applicable.

## Data availability statement

Data included in article/supplementary material/referenced in article.

## Funding source

1.The National Natural Science Foundation Project (Nos.82374445, 82074427 and 82305233); 2. The Key Discipline Construction Project of Shanghai's Three Year Action Plan for Strengthening the Construction of Public Health System (2023–2025) (Nos.GWVI-11.1-24); 3. High-level Chinese Medicine Key Discipline Construction Project (Integrative Chinese and Western Medicine Clinic) of National Administration of TCM (zyyzdxk-2023065); 4. Evidence-based demmotology base sponsored by State Administration of Traditional Chinese medicine; 5. The Dawn Project (Nos.22SG41); 7. The Medical innovation research project of Shanghai 2021 "Science and Technology Innovation Action Plan" (Nos.21Y21920102); 8. the Clinical Research Plan of Shanghai Shenkang Hospital Development Center (Nos.SHDC22022302); 9. Shanghai Oriental Talent Program for Top-notch Project (Nos.BJWS2024046).

## Declaration of competing interest

The authors declare that they have no known competing financial interests or personal relationships that could have appeared to influence the work reported in this paper.

## Figures and Tables

**figs1:**