# Supplementary material for: Efficacy and safety of Janus kinase inhibitors in the treatment of psoriasis and psoriatic arthritis: An analysis of evidence from 2014 to 2022
Source: Heliyon. 2025 Jan 28;11(3):e42084. doi: 10.1016/j.heliyon.2025.e42084 (PMC11848086; doi:10.1016/j.heliyon.2025.e42084)
Supplement: Multimedia component 1 [file mmc1.doc]

**Table S1. Classification of adverse events of included studies**

| **Type** | **Performances** |
| --- | --- |
| Exercise system | Back pain, Osteomyelitis |
| Nervous system | Headache, Abnormal dreams, Fatigue, Nervous system disorders, Fever, Headache, Dizziness |
| Circulatory system | Blood CPK increased, Decreased neutrophils, Peripheral oedema, Blood and lymphatic system disorders, Platelet count decreased, Absolute neutrophil count decreased, WBC count decreased, Vascular disorders, Fall in Hb., Rise in BP |
| Respiratory system | Nasopharyngitis, Pharyngolaryngeal pain, Upper respiratory tract infection, Dry mouth, Nasal congestion, Sinusitis, Bronchitis, Pneumonia, Lower respiratory tract infection, Influenza, Cough, Oral ulcers/Sore mouth, |
| Digestive system | Diarrhoea, Flatulence, Dyspepsia, Abdominal pain, Hyperlipidemia, Nausea, Stomach discomfort, Vomiting, Tooth infection, Gastroenteritis, Gastroenteritis Viral, Periodontitis, AST increased, Hypertriglyceridemia, Gastrointestinal disorders, Constipation, Heartburn, Rise in SGPT >ULN |
| Urinary system | Hematuria, Urinary tract Infection, Burning micturition |
| Skin | Acne, Contact dermatitis, Fungal infection, Furuncle, Herpes simplex, Herpes zoster, Paronychia, Viral rash, Folliculitis, Leg cellulitis |
| Others | Postop wound infection, Infections and infestations, Ear infection, Wound infection, Viral infection, Injury, poisoning and procedural complications, Severe weight loss |
