# Supplementary material for: Efficacy and safety of Janus kinase inhibitors in the treatment of psoriasis and psoriatic arthritis: An analysis of evidence from 2014 to 2022
Source: Heliyon. 2025 Jan 28;11(3):e42084. doi: 10.1016/j.heliyon.2025.e42084 (PMC11848086; doi:10.1016/j.heliyon.2025.e42084)
Supplement: Multimedia component 2 [file mmc2.docx]

**Table S2. Characteristics of included randomized controlled studies**

| **Study** | **Drug** | **Target** | **Usage** | **Diagnosis** | **Control** | **Phase** | **Multicenter (Y/N)** | **Blind (Y/N)** | **Region** | Sex（M/F） | **Enrollment  number** | **Intervention Methods** | | **Sample size (M/F)** | | **Course of treatment** | **Outcome measures** |
| --- | --- | --- | --- | --- | --- | --- | --- | --- | --- | --- | --- | --- | --- | --- | --- | --- | --- |
|  |  |  |  |  |  |  |  |  |  |  |  | **E** | **C** | **E** | **C** |  |  |
| K.A. Papp et al  2016 | Tofacitinib | JAK1 and JAK3 | External use | PSO | vehicle-controlled | 2b | N | Y | U.S.A., Canada, Denmark, Poland | 260/170 | NCT01831466 | E1:2 % (20 mg/g) tofacitinib ointment，BID E2:1 %(10 mg/g) tofacitinib ointment,BID E3:2 % (20 mg/g) tofacitinib ointment，QD E4:1 %(10 mg/g) tofacitinib ointment, QD | **C1:**vehicle, BID C2:vehicle, QD | 71  70 70  74 | 71 74 | 12W | PASI75, PGA-C, ISI, BSA, DLQI, AE |
| K. Papp et al  2015 | ASP015K | JAK3 | Oral | PSO | placebo-controlled | 2a | Y | Y | U.S.A. | 97/27 | NCT01096862 | E1:ASP015K 10 mg, BID E2:ASP015K 25 mg, BID E3:ASP015K 60 mg, BID E4:ASP015K 100 mg, BID E5:ASP015K 50 mg, QD | **C1:**placebo | 19 21 19 17 19 | 29 | 6W | PASI, PGA-C, AE |
| W.C. Ports et al  2013 | Tofacitinib | JAK1 and JAK3 | External use | PSO | vehicle-controlled | 2a | Y | Y | Canada, U.S.A. | 43/28 | NCT01246583 | E1:2 % (20 mg/g) tofacitinib ointment 1,BID E1:2 % (20 mg/g) tofacitinib ointment 2,BID | **C1:**vehicle, BID C2:vehicle, BID | 23 25 | 13 10 | 4W | TPSS, TPA, ISI, PSSM |
| C. Mamolo et al  2014 | Tofacitinib | JAK1 and JAK3 | Oral | PSO | placebo-controlled | 2b | Y | Y | N/A | 125/72 | NCT00678210 | E1:2 mg Tofacitinib, BID E2:5 mg Tofacitinib, BID E3:15 mg Tofacitinib ,BID | C1:Placebo | 49 49 49 | 50 | 12W | DLQI, ISS, SF-36, PDA , PSSM, PtGA |
| S.R. Feldman et al  2016 | Tofacitinib | JAK1 and JAK3 | Oral | PSO | placebo-controlled | 3 | Y | N/A | N/A | 643/258 | NCT01276639 (OPT 1) | E1:5 mg Tofacitinib, BID E2:10 mg Tofacitinib, BID | C1:Placebo | 363 360 | 177 | 52W | DLQI, ISI, JPA, PtGA Patient satisfaction |
|  |  |  |  |  |  |  |  |  |  | 648/312 | NCT01309737 (OPT 2) | E1:5 mg Tofacitinib ,BID E2:10 mg Tofacitinib ,BID | C2:Placebo | 382 381 | 196 | 52W |  |
| K.A. Papp et al  2016 | Baricitinib | JAK1/JAK2 | Oral | PSO | placebo-controlled | 2b | N | Y | U.S.A., Canada, Japan | 197/74 | NCT01490632 | E1:baricitinib 2 mg, QD E2:baricitinib 4 mg, QD E3:baricitinib 8 mg, QD E4:baricitinib 10 mg, QD | C1:Placebo | 32 72 64 69 | 34 | 12W | PASI 75, PASI 50, PASI 90, sPGA, The Worst Itch Numeric Rating Scale score, DLQI, AEs |
| N. Punwani et al  2012 | INCB018424 | JAK1 and JAK2 | External use | PSO | vehicle-controlled | N/A | N | Y | N/A | 20/9 | NCT00820950 | E1: INCB018424 phosphate cream 0.5%, QD  E2: INCB018424 phosphate cream 1.0%, QD E3: INCB018424 phosphate cream 1.5% ,BID E4: INCB018424 phosphate cream 1.5%, BID  E5: INCB018424 phosphate cream 1.5%, BID | C1:vehicle, QD C2:vehicle, QD C3:vehicle, BID C4:calcipotriene 0.005% cream, BID  C5:Betamethasone dipropionate 0.05% cream, BID | 6 6 6 6 5 | 6 6 6 6 5 | 8W | Lesion area |
| R. Bissonnette et al  2016 | INCB039110 | JAK1 | Oral | PSO | placebo-controlled | 2 | Y | Y | Canada, U.S.A. | 32/18 | NCT01634087 | E1:INCB018424 100 mg, QD E2:INCB018424 200 mg, QD E3:INCB018424 200 mg, BID E4:INCB018424 600 mg, QD | C1:Placebo | 9 9 9 11 | 12 | 4W | PASI 75, PASI50, sPGA, BSA, AEs |
| V.J. Ludbrook et al  2016 | GSK2586184 | JAK1 | Oral | PSO | placebo-controlled | 2a | Y | N | U.K., Germany | 35/14 | NCT01782664 | E1:100 mg GSK2586184, BID E2:200 mg GSK2586184, BID E3:400 mg GSK2586184, BID | C1:Placebo | 15  16` 14 | 15 | 12W | PASI 75, PASI 50, PASI 90, PGA, DLQI, VAS, AEs, ECG |
| G.J. Schmieder et al 2018 | PF-04965842 | JAK1 | Oral | PSO | placebo-controlled | 2 | Y | Y | U.S.A.,  Canada | 40/19 | NCT02201524 | E1:200 mg PF-04965842, QD E2:400 mg PF-04965842, QD E3:200 mg PF-04965842, BID | C1:Placebo | 15  16` 14 | 15 | 4W | PASI , BSA, DLQI, PGA, PtGA, ISS, AEs |
| J.Z. Zhang et al  2017 | Tofacitinib | JAK1  and JAK3 | Oral | PSO | placebo-controlled | 3 | Y | Y | China mainland, Taiwan, Korea | 388/144 | NCT01815424 | E1:Tofacitinib 5 mg, BID E2:ofacitinib 10 mg, BID | C1:Placebo, BID | 88 90 | 44 | 16W | PASI 75, PGA, BSA, DLQI, PASI 90, AEs |
| K.A. Papp et al  2016 | Tofacitinib | JAK1  and JAK3 | Oral | PSO | placebo-controlled | 3 | Y | Y | U.S.A., Canada, Colombia, Germany, Hungary, Japan,  Mexico, Poland, Puerto Rico | 1291/568 | NCT01276639 NCT01309737 | E1:Tofacitinib 5 mg, BID E2:ofacitinib 10 mg, BID | C1:Placebo, BID | 745 741 | 373 | 16W | PASI 75, PGA, PSAI 90, AEs |
| H. Bachelez et al  2015 | Tofacitinib | JAK1  and JAK3 | Oral | PSO | placebo-controlled | 3 | Y | Y | U.S.A., Canada | N/A | NCT01241591 | E1: Tofacitinib 5 mg, BID E2: Tofacitinib 10 mg, BID | C1: Etanercept 50 mg, BID C2: Placebo | 329 330 | 335 107 | 12W | PASI 75, PGA, PASI50, DLQI, AEs |
| P. Mease et al  2018 | Filgotinib | JAK1 | Oral | PsA | placebo-controlled | 2 | N | Y | Belgium, Bulgaria, Czech Republic, Estonia, Poland, Spain, and Ukraine | 65/66 | NCT03101670 | E1: Filgotinib 200 mg，QD | C1: Placebo | 65 | 66 | 16W | ACR20, ACR50 , ACR70, DAPSA, MDA, NRS, PASDAS, PASI75, PsARC, LEI, SPARCC |
| A.M. Orbai et al  2020 | Filgotinib | JAK1 | Oral | PsA | placebo-controlled | 2 | Y | Y | N/A | N/A | NCT03101670 | E1:Filgotinib 200 mg, QD | C1: Placebo | 65 | 66 | 16W | PsAID9 , SF-36  Correlation between PsAID9 and SF-36 |
| P.Sharma et al  2018 | Tofacitinib | JAK1/JAK3 | Oral | PsA | positive-controlled | 1, 2 | N | N | Dhaka | 34/27 | N/A | E1:5 mg Tofacitinib, BID | C1:MTX | 29 | 32 | 4W | ACR20, ACR50 , ACR70, SJC/TJC, VAS, ESR, CRP, DAPSA, DAS28, PASI 75, MASE, HAQ-DI, AE |

**Abbreviations:** JAK, Janus Kinase; PSO, psoriasis; PsA, psoriatic arthritis; PASI, psoriasis area and severity index; BSA, body surface area; DLQI, Dermatology Life Quality Index; PGA-C, Physician’s Global Assessment; ISI, itch severity item; PtGA, Patient Global Assessment; ACR, American College of Rheumatology; SF-36, Short Form-36 questionnaire, version 2; DAPSA, disease activity in psoriatic arthritis; AEs, adverse events; TPSS, target plaque Severity score; TPA, target plaque area; PSSM, patient satisfaction with study medication; PDA, pain/discomfort assessment; JPA, joint pain assessment; VAS, visual analog scale; ECG, electrocardiogram; MDA, minimal disease; NRS, numerical rating scale; SPARCC, Spondyloarthritis Research Consortium of activity; LEI, Leeds Enthesitis Index; TJC/SJC, Tender Joint Count/Swollen Joint Count; DAS-28, Disease Activity Score-28; MASES, Maastricht Ankylosing Spondylitis Enthesitis Score; ITT, Intention to Treat; E, experiment; C, control; N/A, Not applicable; M, Male; F, Female; BID, twice daily; QD, once a day.
