# Supplementary material for: Efficacy and safety of Janus kinase inhibitors in the treatment of psoriasis and psoriatic arthritis: An analysis of evidence from 2014 to 2022
Source: Heliyon. 2025 Jan 28;11(3):e42084. doi: 10.1016/j.heliyon.2025.e42084 (PMC11848086; doi:10.1016/j.heliyon.2025.e42084)
Supplement: Multimedia component 3 [file mmc3.doc]

**Table S3. The Cochrane Collaboration’s tool for assessing risk of bias of RCTs**

|  | Random sequence generation | Allocation concealment | Blinding of participants and personnel | Blinding of outcome assessment | Incomplete outcome data | Selective outcome reporting | Other source of bias |
| --- | --- | --- | --- | --- | --- | --- | --- |
| K. A. Papp et al 2016 | + | + | + | + | + | + | + |
| K. Papp et al 2015 | ? | + | + | + | + | + | + |
| W.C. Ports et al 2013 | + | + | + | + | + | - | + |
| C. Mamolo et al 2012 | + | + | + | + | + | + | + |
| S.R. Feldman et al 2016 | ? | ? | ? | ? | + | + | + |
| K.A. Papp et al 2016 | + | + | + | + | + | + | + |
| 1. Punwani et al 2012 | + | + | + | + | + | + | + |
| R. Bissonnette et al 2015 | ? | ? | + | + | + | + | + |
| V.J. Ludbrook et al 2016 | + | + | + | + | + | + | + |
| G.J. Schmieder et al 2017 | + | + | + | + | + | + | + |
| J.Z. Zhang et al 2017 | + | + | + | + | + | + | + |
| K. A. Papp et al 2016 | + | + | + | + | + | + | + |
| 1. Bissonnette et al 2014 | + | + | + | + | + | + | + |
| 1. Bachelez et al 2015 | + | + | + | + | + | + | + |
| P. Mease et al 2018 | + | + | + | + | + | + | + |
| A.M. Orbai et al 2020 | ? | ? | + | + | + | + | + |
| P.Sharma et al 2021 | + | - | - | - | + | + | + |

The dark green cells (+) indicate a low risk of bias. The red cells (-) indicate a high risk of bias. The yellow cells (?) indicate an uncertain risk of bias. n/a, not applicable
